# Notes of Progress in the Study of the Pathology of the Nervous System

**Published:** 1874-05-15

**Authors:** Plym. S. Hayes


### NOTES OF PROGRESS IN THE STUDY OF THE PATHOLOGY OF THE NERVOUS SYSTEM.

A PART OF THE ANNUAL REPORT OF THE SECTION ON PATHOLOGY, OF THE CHICAGO SOCIETY OF PHYSICIANS AND SURGEONS. PREPARED BY PLYM. S. HAYES, M.D.

PATHOLOGY OF THE PERIPHERAL NERVES.—M. Verneuil, in four articles (*Gazette Medicale de Paris*), gives the history, illustrated by cases, of what he terms traumatic herpes. This is a vesicular eruption, resembling in character herpes or zona. It is often spontaneous, or consecutive to an injury. He concludes that herpes may exist as an independent intercurrent affection during the evolution from an injury; and that it may also certainly result from the wound, and be really of traumatic origin. Three forms of traumatic herpes may be distinguished—peripheral, contiguous, and distant herpes. The disorder may follow a wound of a nerve track, a ganglion, or of the peripheral ends of the nerves. The herpes may relapse; it may coincide with erysipelas, and simulate the vesicular variety of that disease.

We learn from the *Medical Times and Gazette*, that Prof. Eberth, of Zurich, proposes a new explanation of the occurrence of inflammation of the cornea, dependent upon lesion of the fifth pair of nerves. He maintains that the trauma is the mediate, rather than the immediate, cause of the corneal inflammation. The pathological condition of the nerve induces changes in the eye favorable to the retention of bacteria in the cornea, and the subsequent formation of bacteric masses, and attendant inflammation.

The most superficial puncture of the affected spot causes a rapid extension of the disease. The occurrence of the keratitis is, however, dependent upon the degree and extent of the desication, the amount of protrusion, and the size of the ocular aperture.

Dr. Edward Hansen has published (in the *Hospitals-Tidende*) a paper on "Intermittent Neuralgic Vesicular Keratitis depending on Traumatic causes." This disease takes its origin from injury to the peripheral extremities of the nerves, probably those of the corneal epithelium. These nerves, undoubtedly, exercise an important influence on the vitality of the epithelial cells. The origin of this malady is always a wound, in the shape of a scraping of the epithelium.

Prof. Erasmus Wilson, in a course of six lectures on Dermatology (reported in the *Lancet*), mentions the following implications of the peripheral nerves in leprosy. The pathological complications in this disease were stated to be the development of a cachetic condition, attendant on a colloid metamorphosis infiltrating the tissues and causing their ultimate destruction. The colloid material is deposited in the skin, obliterating the papillary layer. The corium and connected structures, including vessels and nerves, are thus destroyed by the infiltration of tubercular masses. This deposit invades the neurilemma, separates the tubules, and soon leads to their atrophy.

Dr. H. V. Carter, Bombay army, has given a more extended account, in an article on the pathology of leprosy of the nerve lesions due to this disease. This author concludes that there is, prior to the more visible changes of leprosy, a progressive impairment of the functions of the cutaneous nerves and branches. The structural alterations of the nerves he considers to be the characteristic lesion of leprosy. He agrees with Wilson, that the subsequent histological changes are due to the infiltration, or deposition, of morbid materials in the skin and subjacent tissues. This deposit appears in the nerves, between the nerve tubules, and within the sheath. The envelope of connective tissue of the nerves is hardly changed. This deposition continues until the tubules are separated, compressed, emptied of their contents, and eventually destroyed. The tactile corpuscles disappear before the other less sentient corpuscles. The brain and spinal cord are wholly free from such deposit. The cutaneous nerves are implicated, chiefly and primarily, in that part of their course between the deeper fascia and the limbs and trunk. When the deeper seated nerves are affected, it appears to be only in their sensory elements; and those nerves are involved which are in continuity with cutaneous nerves. Motor paralysis is seldom marked, even in those who have lost hands and eet by the disease.

The author considers that there are trophic nerves in connection with the sensory, and that, through these, nutrition may be affected directly by the nervous system. It is submitted that all of the essential phenomena of leprous diseases may be traced directly or remotely to the characteristic nerve lesion.

Vulpian (*Journal of Anatomy and Physiology*) demonstrates that the gray matter of the spinal cord, medulla oblongata, and the corresponding parts of the pons varolii, exert, through the nerves of motion, a trophic influence on the muscles, as well as on the nerves themselves. This is shown by the atrophy of muscles, when their motor nerves are severed and they are therefore no longer in contact with their trophic centers. This obtains even when the severed motor nerve contains no sympathetic fibres at the place of division. This shows that the trophic influence is carried to the muscles through their motor fibers. The muscles atrophy when the anterior horns of the gray substance of the spinal cord degenerate, and the sympathetic is not involved.

Drs. Bizzozero and Golgi (*Medizinische Jahrbucher*) briefly give a summary of the researches of Montegazza and Vulpian, as well as of the views of Fasce and Erb, on the changes that take place in muscles after the section of the nerve distributed to them. The remainder of the article is devoted to their own researches on the changes which take place in muscular tissue after section of nerves. Their principal experiment was that which occupied the longest time from the section of the nerve (sciatic) until the death of the rabbit (eleven months afterwards), and subsequent microscopical examination. "Microscopical investigation showed, in the superficial muscles of the thigh, isolated rows of fat granules, which seemed to correspond to the course of the nerve fibers. In the deep-seated muscles of the thigh, the muscular fibers were found attenuated here and there; the transverse striæ were not well marked; and between the muscular bundles of the first and second order were seen numerous large zones of fat cells. At other parts, the muscular substance of single fibers was partly torn in pieces, and partly replaced by fat cells.

"The superficial muscles of the leg presented, very markedly, those appearances which are generally represented as the result of section of nerves; that is to say, multiplication of the nuclei of the muscle corpuscles, withering of the fibers, increase of the interstitial connective tissue, and profuse deposit of fat cells between the muscular fibers.

"In the deep-seated muscles of the lower extremity, which were yellow and lardaceous, no traces of muscular tissue could be found; it seemed to have been converted into fatty tissue, which resembled that of the panniculus adiposus. On transversal section, the fat cells presented round or irregular margins, and were arranged as in a mosaic. On longitudinal section, they were seen to be arranged in rows corresponding in direction to the fibres and muscles.

"In other investigations, of shorter duration, we met with fat cells only between the larger bundles. In one case, which had been observed for two months only, the fat cells were scattered between the bundles. In one case, observed for four months, we failed to find any trace of fatty deposit.

"These observations appear to us to be of some importance, as they indicate the causes of certain varieties in diseased conditions, as pseudo-hypertrophic paralysis, in the advanced stages of which the muscular fibres are found separated by fatty tissue."

Dr. Petrow (*Virchow's Arch.*) gives the following hystological changes that take place in the nervous system of the great sympathetic, in cases of acquired constitutional syphilis: 1. Modifications of the protoplasma of nervous cells, which become loaded with brilliant pigmentary corpuscles, increasing with the age of the disease, and often accompanied by colloid transformation of the cells; the cells of the endothelium surrounding the nervous cells frequently undergo the same gelatiniform transformation, and cannot then be distinguished from the nervous cells. These changes can exist without the interstitial connective tissue being impaired. 2. Modifications of the interstitial connective tissue, with hyperplasia of the fibers, constituting large, irregular fasciculi, which push aside and compress the nervous cells and fibers. The cells are then atrophied, irregular, and dotted with pigment, whilst the fibers are flattened, and their myeline shows slight granulations.

The *Gazette Medicale de Paris* contains the summary of a case of injury of the sciatic nerve, followed by epilepsy. This is one of the few cases of injury to this nerve which has been reported as being followed by epilepsy. It is interesting, in view of Dr. Brown-Sequard's experiments on animals for the artificial production of epilepsy by severing the sciatic nerve. The outlines of the case are follows: The patient, a soldier, received a gun-shot wound of the left thigh, Nov. 7th, 1870. The bullet, although visible, was so deeply imbedded that it could not be removed. In the course of six or seven months he began to have convulsive attacks, which were quite violent. The wound was then cicatrized, and the projectile could no longer be felt on palpation. These attacks then grew less and less frequent; but his health gradually began to fail. An operation was subsequently performed and the nerve exposed, and found surrounded by a cicatrix of connective tissue. Subsequent to this operation there have been none of the convulsive attacks.

Dr. Matthew Duncan (*Brit. Medical Jour.*) gives the details of a case of epilepsy produced by digital impression in the cranium of a fœtus during birth. The labor, a difficult one, occurred in a lady who had married late in life. Manipulation was required, and pressure made on the left parietal bone. The bone yielded to the pressure, and a digital impression was produced, which persisted, and was not entirely flattened out until two weeks after the birth of the child. On the third day after birth, the nurse noticed a twitching of the face and superior extremities. These gradually grew worse for about three weeks. The disease eventually yielded to treatment.

INTRASPINAL LESIONS.—The *British Medical Journal* contains a case reported by Dr. Nieden, of a traumatic lesion of the spinal cord, corresponding to the first and second dorsal vertebræ. The two lower extremities, and the trunk as high as the second intercostal space, were paralyzed completely to motion and sensation. The temperature gradually fell from 95° to 80.6° F., when death occurred, ten days after the accident. During this time there were two periods of increment. The pulse and respirations bore an almost constant proportion to the diminution of temperature. The patient was conscious, even to the last.

The following salient points have been taken from an article on "Intraspinal Hæmorrhages," by P. Hayem (*Archives Generales de Medicine*). After dividing these hæmorrhages into extrameningeal, intrameningeal, and subarachnoid, he says that, of all, the extrameningeal is the most frequent. The blood may be effused the whole extent of the space between the spinal canal and dura mater. The clots vary much in size and consistence. The amount of effusion in this locality never seems to be sufficient to compress the cord. Interarachnoid hæmorrhage usually occupies the whole height of the membranes, and generally compresses the cord. The subarachnoid variety is very rare. After having pricked an intraspinal vein in a dog, he found the three varieties of hæmorrhage were produced—varieties which were met with simultaneously in the cases recorded. According to this author, spinal hæmorrhage has nothing parallel to cerebral hæmorrhage. In fact, spinal hæmorrhage is so generally accompanied by inflammation, that it might be properly termed hæmatomyelitis. At times the blood is contained in an anfractuous cavity; but more generally is it intimately mixed with nerve substance. The effusion occurs in the gray substance of the cord, the white portion interposing an insurmountable barrier. In the great majority of cases, the disorganization of the gray substance is entirely out of proportion to the amount of hæmorrhage. The whole extent of the cord may be affected when the hæmorrhage is not more than a centimetre in breadth. At times, the hæmorrhagic centers may be disseminated throughout the extent of the cord. M. Lionville found, in the vessels, capillary aneurisms similar to those found in the brain. The vessels are usually thickened, and their sheaths enlarged, distended and filled with yellow, fatty granulations.

M. Rosenthal observes, that the chief pathologico-anatomical lesions that are met with after death in the spinal or essential palsy of children, are atrophy and malformation of the anterior cornua of the cord. According to this author, the changes observed in the cells of the gray substance are not the primary lesion. These changes depend, principally, on the enlargement and thickening of the blood-vessels observed by him. These changes in the blood-vessels antedate the destruction of the gray substance, and stand in the relation of cause to effect.

INTRACRANIAL LESIONS.—J. Lockhart Clark (*British Med. Jour.*) gives the details of a case of progressive muscular atrophy, accompanied by muscular rigidity and contraction of the joints, with *post-mortem* examination of the brain and spinal cord. The duration of the disease, from the first appearance of vertigo until death ensued, was twenty-eight years. The general history was that of a typical case. The examination showed that the cerebral convolutions were thickly interspersed with corpora amylacea, and that many of the blood-cells of the white substance were enlarged. The cells of the gray substance were not altogether healthy. The pons varolii was below the average size. Its blood - vessels were somewhat dilated. In it the corpora amylacea were thickly interspersed. The medulla oblongata was about one-fifth below the average size. All its nuclei were smaller than normal, their cells having undergone a pigmentary degeneration. The spinal cord was one-fourth less than the natural size. In all of its parts the gray substance was in a pathological condition, from various lesions and degenerations. The nerve cells of the anterior gray substance throughout the length of the cord had undergone degeneration; some of the cells having undergone pigmentary degeneration, others having fallen into granular heaps. Of those that remained, their processes were either lost or reduced considerably in size.

M. Lionville relates (*Gaz. des Hopitaux*) a case of complete paralysis, with only slight impairment of sensation. The patient was found on the street insensible. The urine was drawn off, and found loaded with albumen and sugar. There was no indication of Bright's disease. The necropsy showed that a large portion of the pons Varolii had been invaded by the hæmorrhage, and the upper part of the wall of the fourth ventricle was affected.

Zenker has, for several years past, devoted himself to the study of the pathogeny of spontaneous cerebral hæmorrhage. During this time, in every case that he has examined with sufficient care, he has found miliary aneurisms present, not only in the neighborhood of the clot, but in other parts of the brain. All of the arterial coats bound these vascular dilations, thereby forming true aneurisms. The inner coat of the artery first becomes ruptured, thus creating a dissecting aneurism. This state of things may continue for some time. A retrograde action may be set up, so that nothing eventually remains but a little pigmentary tubercle. Lastly, the aneurismal wall may rupture, and give rise to cerebral hæmorrhage. So far do MM. Charcot and Bouchard, who first discovered the frequent presence and pathogenic influence of these aneurisms, agree with Zenker. As to the immediate cause of the formation of the aneurisms, they differ. Zenker claims that these capillary aneurisms are preceded, as all true aneurisms of the larger arteries are, by sclerosis of the inner tunic of the cerebral arterioles.

Even if miliary aneurisms can exist without change in the arteries at the base of the brain, microscopical investigations show that the arterial branches in the neighborhood of the aneurisms have undergone a change in their inner coats. This change consists of an irregular thickening and sclerosis, and sometimes fatty degeneration.

Dr. Lidell, in an article in the *American Journal of Medical Sciences,* has collected the histories of a number of cases of thrombosis of the cerebral arteries. After making remarks on each case, and dividing them into eight classes, he gives the etiology, anatomical appearances, symptoms, diagnosis, and treatment, of this disease.

In answer to the self-asked question, Does thrombosis ever begin in the minute arteries of the brain, in old people? he replies that "there does not appear any good reason why thrombosis should not sometimes have its starting-point in the cerebral capillaries, as well as in the capillaries of the extremities of aged persons." The anatomical changes in the brain substance are due to anæmia, from the greatly diminished supply of nutrient blood. These changes consist in an exsanguinated appearance, and diminished consistence of the brain substance. Anæmia is not always present, for in some cases the portion of the brain implicated exhibits an undue congestion, the white substance, when cut, becoming quickly bedewed with blood. The author thinks that want of exsanguination has its origin in the vaso-motor paralysis, and dilation of the cerebral vessels, a state which preceded and determined the thrombosis. The softening of the brain substance is strictly a necrosis, analogous to gangrene in the extremities.

The reason assigned for the brain not exhaling a gangrenous odor, is because of its complete exclusion from the air. The necrosed portion varies in size from that of a bean to a goose's egg. There was found, on microscopical examination of the necrosed substance, only the remains of nerve filaments, granular cells that have undergone fatty degeneration, and masses of detritus. There were no granule cells or granular masses, such as are found in softening from exudation. Should collateral circulation be soon established, the anæmia is in a measure removed, the paralyzed limbs regain their lost functions, and the other signs of cerebral disturbance pass away. Even after collateral circulation has been established, and the paralysis and brain symptoms abated in a measure, the cerebral lesion may be sufficient to produce death.

Dr. L. Waldenburg reports a case of congenital aphasia. The child at the time of the report was six years old. The mother had an attack of right hemiplegia, accompanied by aphasia, when three months pregnant. The child was born at term, with the symptoms of right hemiplegia. He has recovered almost entirely from the effects of the paralysis; is not deaf, and is quite intelligent. The examination of his mouth and vocal organs was negative, rather than positive, in affording an explanation of the cause. He never tries to produce articulate sounds. The Doctor thinks this case tends to disprove that both cerebral hemispheres are capable of educated speech.

Dr. J. L. Smith, in an article in the *American Journal of Medical Sciences,* gives the necropsy of seventy-six cases of cerebro-spinal fever. The following is a condensed statement of some of the most important pathologico-anatomical facts that he has stated in his conclusions. The amount of fibrin in the blood is increased, in cases that are not speedily fatal. In those who die in the stage of acute inflammatory congestion, the cranial sinuses are found engorged with blood, and contain soft, dark clots. In those cases which end fatally in a few hours, there is usually no other lesion than a hyperæmia of the meninges. In cases of longer duration, there is an exudation of serum and fibrin from the vessels into the meshes of the pia mater, and over the surface of the brain beneath this membrane. Pus cells are mixed with the fibrin. At times the pus cells are so few as to be discovered only by the microscope; at other times the pus is in excess of the fibrin, and is readily detected by the unaided eye. The arachnoid loses its transparency and polish, and presents a cloudy appearance over a greater or less extent of its surface. The exudation of serum, fibrin and pus is more abundant in the spaces around the course of the vessels, over and around the optic commissure, the pons Varolii, the cerebellum, medulla oblongata, and the Sylvian fissures.

The quantity of serous exudation varies greatly with the case. If this exudation is of considerable quantity, the convolutions may be flattened, and the amount of blood circulating in the brain be less than normal. Cerebral softening occurs in certain cases. This softening is usually local, rather than general. The ventricles contain, in some cases, serum alone; in others, the serum is turbid, containing flocculi of fibrin, or fibrin and pus. In advanced cases, with abatement of the inflammation, the serum is obviously first absorbed, while the fibrin and pus are more slowly removed by fatty degeneration, and liquefaction. The author thinks that the remains of the fibrinous exudation may be found in persons who have recovered from this disease, although he has not verified this statement by *post-mortem* examination. The changes that take place in the spinal cord and membranes are similar to those found in the brain and its membranes.

